# Fractional CO_2_ laser in the treatment of nail psoriasis: how can it help?

**DOI:** 10.1007/s00403-023-02574-w

**Published:** 2023-02-21

**Authors:** Ahmed Abdelfattah Afify, Maha Adel Shaheen, Mahmoud Gamal El-Banna

**Affiliations:** Dermatology, Venereology and Andrology Department, Faculty of Medicine, Ain Shams University, Cairo, Egypt

**Keywords:** Fractional, Laser, Nail, Psoriasis, How-Help

## Abstract

Treating nail psoriasis is often a time-consuming challenge with an unsecure outcome. Response to the treatment is variable and relapses are common. Systemic treatments have multiple systemic side effects and lack of the patient compliance makes intra-lesional therapies not the best choice for treatment of nail psoriasis. We aimed to evaluate and compare the efficacy and side effects of methotrexate versus calcipotriol plus betamethasone two-compound formula when applied topically to psoriatic nails after fractional CO_2_ laser. This comparative pilot study included 20 patients with nail psoriasis. One side was treated with fractional CO_2_ laser followed by the application of topical methotrexate (Group A) and the other side with fractional CO_2_ laser followed by topical (Calcipotriol 0.05 mg/gm + Betamethasone 0.5 mg/gm) (Group B). 4 sessions were done, once every 2 weeks. There was a high statistical significant decrease in total NAPSI score in group A at 1(*P* = 0.000) and 2 months (*P* = 0.000). There was a high statistical significant decrease in total NA*P*SI score in group B at 1(*P* = 0.001) and 2 months (*P* = 0.001). There was no statistical significant difference regarding total NA*P*SI score between both group A and B at 0 (*P* = 0.271), 1(*P* = 0.513) and 2 months (*P* = 0.647). Combined fractional CO_2_ laser with either topical MTX or topical betamethasone plus calcipotriol two-compound formula is effective treatment for nail psoriasis.

## Introduction

Psoriasis is a chronic immune-mediated disease that results from a genetic predisposition combined with environmental triggers [1].

Psoriatic involvement of the nail affects up to 50% of psoriatic patients during their lifetime. It can occur in both children and adults and is strongly associated with psoriatic arthritis. Nail psoriasis exhibits different types of lesion depending on the affected part of the nail unit. Nail bed psoriasis presents as “oil drop” discoloration, splinter hemorrhages, subungual hyperkeratosis, and onycholysis, whereas nail matrix psoriasis usually presents as pitting, leukonychia, erythema of the lunula, and crumbling [2]. Koebner phenomenon likely occurs in 25% of patients with psoriasis after various traumatic injuries, and nail psoriasis has been reported to be triggered by surgical reconstruction of syndactyly [3].

Such disfigurment is considered to be a significant cosmetic handicap and the impact on quality of life is very high. Nail psoriasis is often refractory to traditional treatments and it is difficult to find an effective agent with absent or minimal systemic side effects [4].

Carbon dioxide (CO_2_) laser is one of the most widely used lasers in the dermatology field. With its wavelength in the mid-infrared at 10,600 nm, CO_2_ laser energy is well absorbed in water. As skin contains a very high water percentage, this makes the CO_2_ laser ideal for precise, safe ablation with good hemostasis [5].

Methotrexate (MTX) therapy for psoriasis has been revised several times since 1972 and has been approved by the FDA. MTX inhibits di-hydro-folate reductase competitively, reducing metabolism of di-hydro-folic acid to tetra-hydro-folic acid which results in suppression of the intracellular synthesis of various folic acid derivatives that play an important role as a cosubstrate in the transport of C1 units, as a consequence, synthesis of purine, thymine, and DNA is disturbed and epithelial hyperplasia is limited [6].

Despite the marked impact of nail psoriasis on quality of life, few studies have explored the efficacy of individual therapeutic options. Injectable therapies including triamcinolone acetonide and MTX have been reported to be effective in limited reports. Patients can be treated with an injection of MTX (0.1 mL of a 25 mg/mL solution) into the nail bed but the injection is painful [7]. Trans-ungual MTX delivery can be enhanced by the fractional laser ablation [8, 9].

Combination of a vitamin D analog and a corticosteroid into a two-compound topical formulation has increased efficacy compared with either drug administered alone. Once-daily application of such a product would likely improve adherence in the treatment of nail psoriasis but the absorption of this topical preparations is minimal, thus limiting its efficacy [10].

In this context, the current study aimed to evaluate and compare the efficacy, safety, and side effects of fractional CO_2_ laser and MTX with fractional CO2 laser and calcipotriol plus betamethasone two-compound formula in the treatment of nail psoriasis.

## Patients and methods

### Participants

This comparative pilot study included 20 patients with nail psoriasis recruited from the dermatology out-patient clinic of Ain–Shams University Hospitals, during the period from March 2019 till October 2019. The study was approved by Research Ethical Committee of Ain Shams University (FMASU MS 71/2019). Patients > 18 years with cutaneous psoriasis plus classical psoriatic nail lesions and bilateral finger or toe nail affection were included. Patients complaining of other diseases causing nail dystrophy, patients with previous topical, or systemic anti-psoriatic agents within 3 months prior to the study, patients with a history of Koebner phenomenon and patients with pustular and erythrodermic psoriasis were excluded.

### Clinical evaluation

Each participant was subjected to the following:

- Informed written consent.
- Full history taking including: personal history, history of psoriasis including age of onset, duration and course, onset of nail affection, history of current or previous systemic/topical therapy for psoriasis/nail psoriasis, and family history of psoriasis.
- Complete general examination.
- Dermatological examination including: distribution and morphology of any skin lesions.
- Nail examination: to diagnose nail psoriasis and exclude other causes of nail changes.
- KOH test was done for patients with query onychomycosis vs nail psoriasis and patients with positive KOH test were excluded.
- NAPSI score and photo documentation using an Iphone 6 s 12 mega pixels camera (Apple Co, USA): was done at base line, 1 month and 2 months after the first treatment session.

### Treatment protocol

Laser sessions were performed once every 2 weeks to all the affected nails for a maximum of 2 months (4 sessions). All affected nails were subjected to topical anesthetic gel (Lidocaine 2% ®, Alexandria Co, Egypt) for about 30 min before each session.

**Fig. 1:**
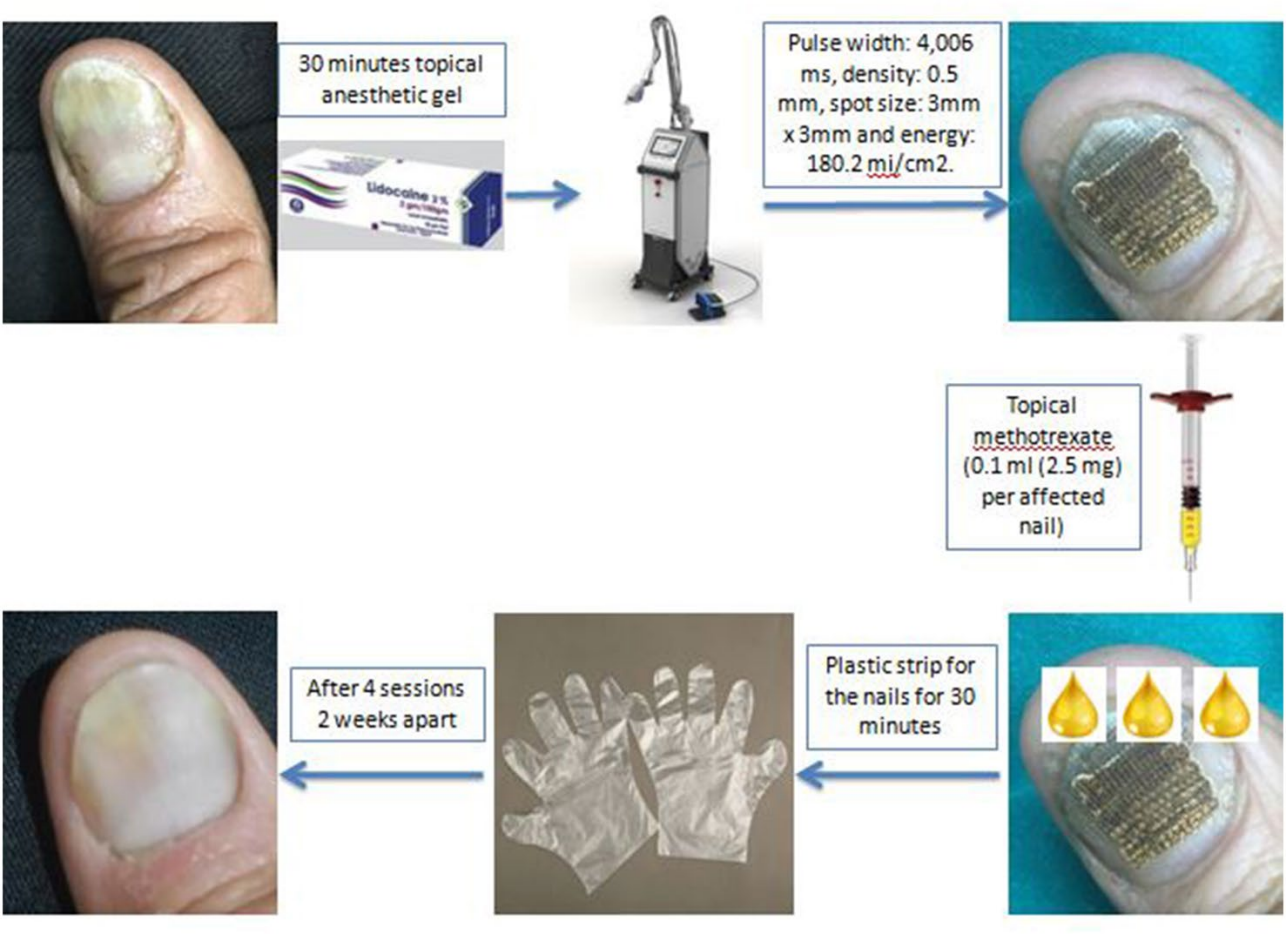
A schematic representation for the steps of the procedure

Before the first session, we decided randomly to treat: One side with: fractional CO_2_ laser (one pass) followed by the application of topical methotrexate (0.1 ml (2.5 mg) of a 25 mg/ml solution per affected nail) (Unitrexate ®, Hikma Co, Egypt). MTX was applied one drop by the insulin syringe for every affected nail, and the patient was fixing his hand on a table and we waited for 3–5 min till this drop is absorbed; then we put the next drop and so on till 0.1 ml was applied for every nail; then we put a plastic strip for the nails for 30 min to ensure complete absorption (Group A) and the other side with: fractional CO_2_ laser (one pass) followed by the application of topical (Calcipotriol 0.05 mg/gm + Betamethasone 0.5 mg/g) (amount of fingertip per affected nail) (Dicalderm ®, Marcyrl Co, Egypt) (Group B). Then we continued with the same treatment protocol during the 3 consecutive sessions with each patient. The fractional CO_2_ laser device used in this study was BISON Fire-Xel Fractional CO_2_ laser (made in Korea), with the following parameters, pulse width: 4,006 ms, repeat delay: single, density: 0.5 mm, spot size: 3 mm × 3 mm and energy: 180.2 mj/cm^2^ (Fig. 1).

### Assessment and evaluation

Assessment of treatment response was done by comparing NAPSI score at base line (0), 1 month and 2 months after the onset of the treatment course. Safety was assessed through detecting any side effects. At the end of the study, subjects were asked to evaluate improvement through scoring their satisfaction using a questionnaire. Patients were asked the following questions: Are you satisfied with this treatment (completely satisfied, satisfied, partially satisfied and not satisfied)? Have you noticed other associated changes? [11].

### Statistical methodology

Data were collected, revised, coded, and entered to the Statistical Package for Social Science (IBM SPSS) version 23. The quantitative data were presented as mean, standard deviations, and ranges when parametric while with non-parametric distribution were presented as median with inter-quartile range (IQR). Also qualitative variables were presented as number and percentages.

The comparison between groups regarding qualitative data was done using chi-square test and/or Fisher’s exact test when the expected count in any cell was less than 5. The comparison between two independent groups with quantitative data and non-parametric distribution was done using Mann–Whitney test. The comparison between two paired groups with quantitative data and non-parametric distribution was done using Wilcoxon rank test. The comparison between more than two paired groups with quantitative data and non-parametric distribution was done using Friedman test. Then adjusted Wilcoxon rank test was used for pairwise comparison when the Friedman test was significant. Spearman correlation coefficients were used to assess the correlation between two quantitative parameters in the same group. *P*-value > 0.05: Non-significant (NS), *P*-value ≤ 0.05: Significant (S) and *P*-value ≤ 0.01: Highly significant (HS).

## Results

Included patients were 13 females (65%) and 7 males (35%) with age ranging from 19 to 60 years (mean 40.30 years ± SD 14.43 years). Duration of the disease ranged from 2 to 20 years with Median (IQR): 5 years (3–8.5). Family history of psoriasis was positive in 15% of cases. 78 psoriatic nails were treated (4 toe nails and 74 finger nails). 2 toe nails and 38 finger nails were treated with fractional CO_2_ laser plus topical methotrexate. 2 toe nails and 36 finger nails were treated with fractional CO_2_ laser plus topical calcipotriol plus betamethasone two-compound formula (Table 1). The total NAPSI score for all nails before the start of treatment was 314, after 1 month was 203 and after 2 months became 122. The total NAPSI score in group A before treatment was 172, after 1 month was 109 and after 2 months became 63. The total NAPSI score in group B before treatment was 142, after 1 month was 94 and after 2 months became 59.

**Table 1:** Demographic characteristics of the studied cases

		Total no. = 20
Age (years)	Mean ± SD	40.30 ± 14.43
	Range	19–60
Sex	Female	13 (65.0%)
	Male	7 (35.0%)
Psoriasis duration	Median (IQR)	5 (3–8.5)
	Range	2–20
Family history	Negative	17 (85.0%)
	Positive	3 (15.0%)
Number of nails treated with fractional CO_2_ laser plus topical methotrexate	2 toe nails	38 finger nails
Number of nails treated with fractional co2 laser plus topical betamethasone/calcipotriol	2 toe nails	36 finger nails

There was no statistical significant relation between total NAPSI score and the age (*P* = 0.755), the duration of the disease (*P* = 0.347) (Table 2) and gender of the studied cases (*P* = 0.082) at baseline (Table 2).

**Table 2:** Correlation between total NAPSI score, age, psoriasis duration and sex at baseline

		Age		Psoriasis duration	
		r	*P*-value*	r	*P*-value*
Total NAPSI score at baseline		– 0.051	0.755	0.153	0.347
Total NAPSI score at baseline	Median (IQR)	Range	Test value	*P*-value**	
Sex	Female	7 (4–13)	3–20	-1.738	0.082
	Male	7 (4–9)	4–11		

^*^Spearman correlation coefficient

^**^Mann–Whitney test

**Fig. 2:**
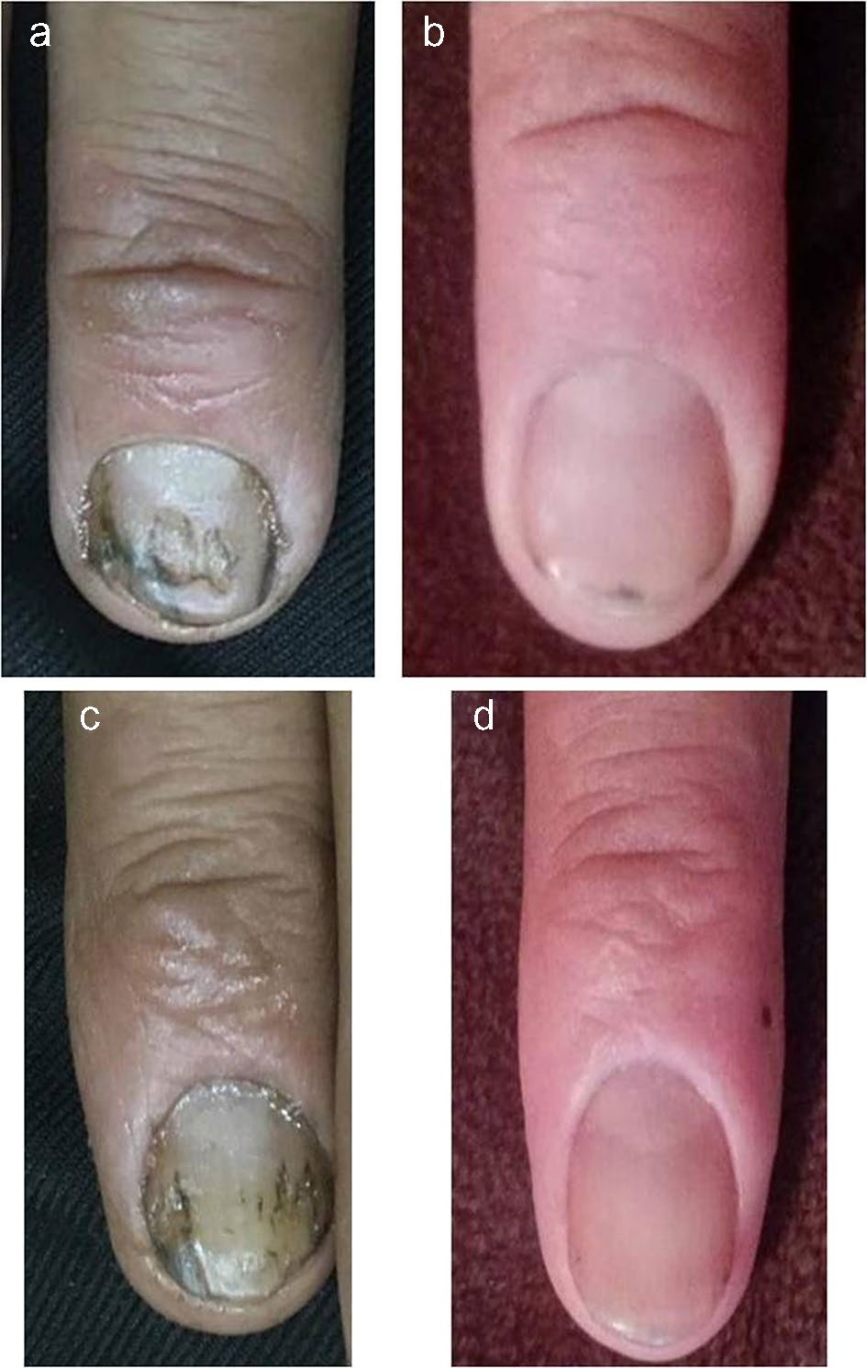
45-year-old man with nail psoriasis in both hands of one-year duration. Right hand was treated by (fractional CO_2_ laser + topical methotrexate) (group A). **a** Before treatment, NAPSI score was 8. **b** After treatment, NAPSI score was 2. There was a marked improvement in nail pitting. Left hand was treated by (fractional co2 laser + (calcipotriol + betamethasone) (group B). **c** Before treatment, NAPSI score was 7. **d** After treatment, NAPSI score was 1. There was a marked improvement in nail pitting and oil drop sign. The patient was completely satisfied

**Fig. 3:**
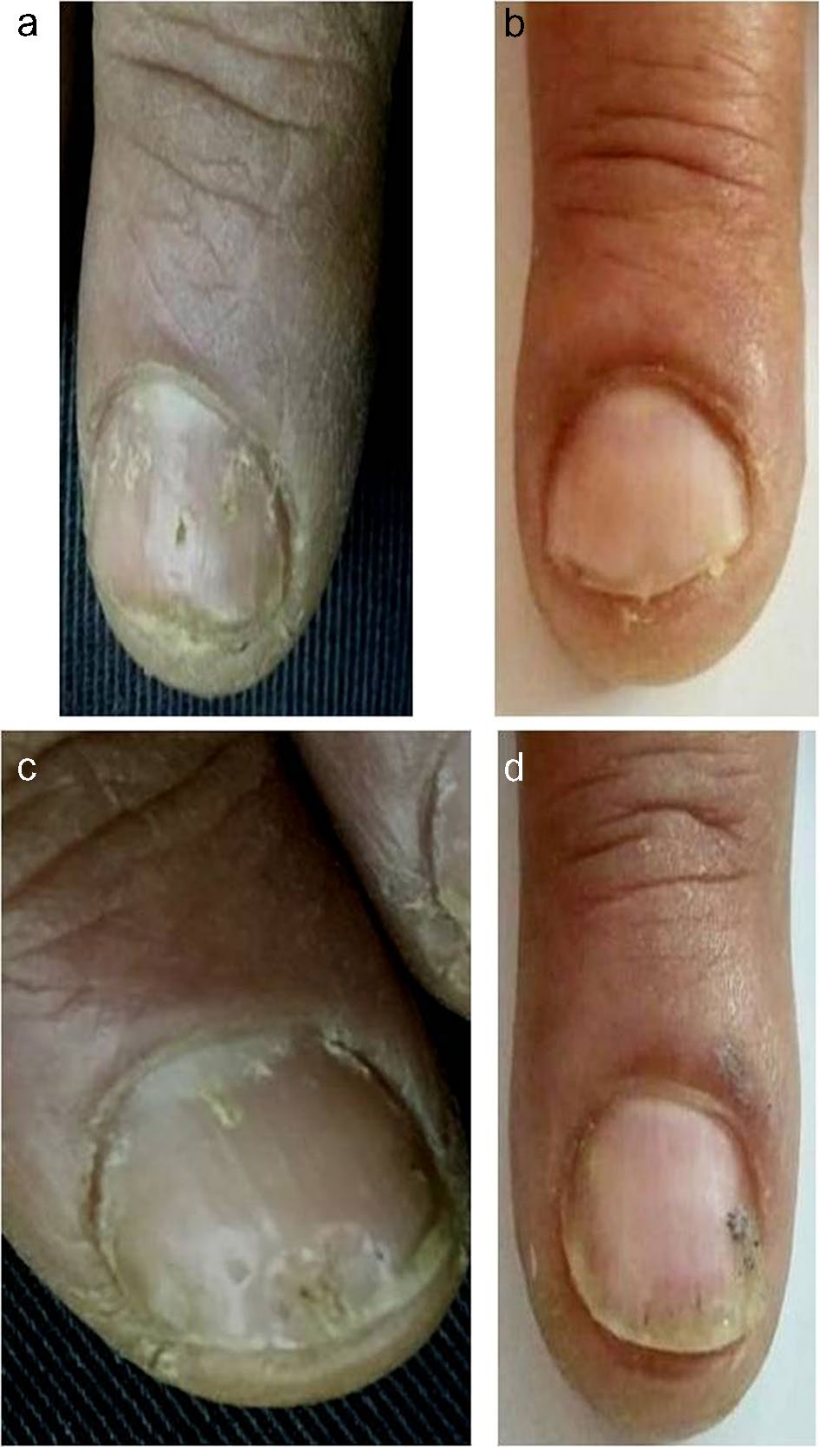
60-year-old man with nail psoriasis in both hands of 3-year duration. Right hand was treated by (fractional CO_2_ laser + topical methotrexate) (group A). **a** Before treatment, NAPSI score was 8. **b** After treatment, NAPSI score was 1. There was a marked improvement in nail pitting. Left hand was treated by (fractional CO_2_ laser + (calcipotriol + betamethasone) (group B). **c** Before treatment, NAPSI score was 4. **d** After treatment, NAPSI score was 2. There was a marked improvement in nail pitting and oil drop sign. The patient was completely satisfied

**Fig. 4:**
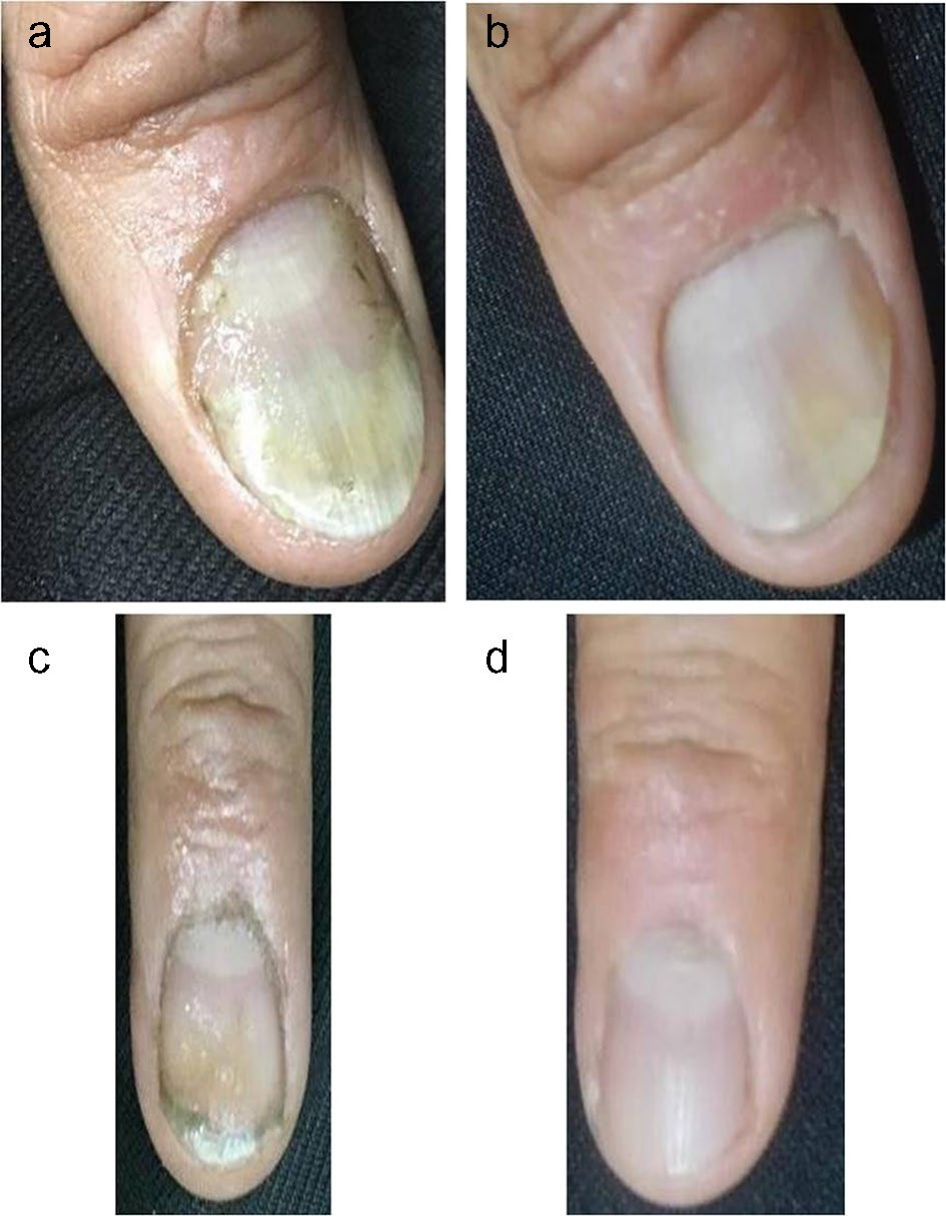
25-year-old man with nail psoriasis in both hands of one-year duration. Right hand was treated by (fractional CO_2_ laser + topical methotrexate) (group A). **a** Before treatment, NAPSI score was 2. **b** After treatment, NAPSI score was 0. There was a marked improvement in onycholysis. Left hand was treated by (fractional CO_2_ laser + (calcipotriol + betamethasone) (group B). **c** Before treatment, NAPSI score was 2. **d** After treatment, NAPSI score was 0. There was a marked improvement in oil drop nail symptom. The patient was completely satisfied

There was a statistical significant decrease in median (IQR) nail matrix NAPSI score in group A at 1 (*P* = 0.011) and 2 months (*P* = 0.012). There was a high statistical significant decrease in median (IQR) nail bed NAPSI score in group A at 1 (*P* = 0.003) and 2 months (*P* = 0.002). There was a high statistical significant decrease in total NAPSI score in group A at 1(*P* = 0.000) and 2 months (*P* = 0.000) (Table 3). There was a high statistical significant decrease in nail matrix NAPSI score in group B at 1 (*P* = 0.007) and 2 months (*P* = 0.007). There was a high statistical significant decrease in nail bed NAPSI score in group B at 1 (*P* = 0.003) and 2 months (*P* = 0.003). There was a high statistical significant decrease in total NAPSI score in group B at 1(*P* = 0.001) and 2 months (*P* = 0.001) (Table 4). (Figs. 2, 3, 4).

**Table 3:** Median (IQR) nail matrix, nail bed, and total NAPSI score at 0, 1, and 2 months in group A

Month/s		Group A	Test value	*P*-value	Group A	Test value	*P*-value	Group A	Test value	*P*-value
		Total Nail matrix score			Total Nail bed score			Total NAPSI score		
0	Median (IQR)	9 (6–13)	–	–	4 (2–8)2–15	–	–	8 (3.5–13) 2–16	–	–
	Range	3–16								
1	Median (IQR)	4.5 (3–10)	-2.558	**0.011**	2 (1–5) 0–7	-2.955	**0.003**	5 (2.5–7) 1–16	-3.531	**0.000**
	Range	2–16								
2	Median (IQR)	1 (1–9)	-2.524	**0.012**	0 (0–2) 0–6	-3.074	**0.002**	1 (0–4) 0–16	-3.627	**0.000**
	Range	0–16								

^*^Wilcoxon test

**Table 4:** Median (IQR) nail matrix, nail bed, and total NAPSI score at 0, 1, and 2 months in group B

Month/s		Group B	Test value	*P*-value	Group B	Test value	*P*-value	Group B	Test value	*P*-value
		Total Nail matrix score			Total Nail bed score			Total NAPSI score		
0	Median (IQR)	5 (4–9)	–	–	3.5 (2–6) 2–9	–	–	5 (4–9) 4–20	–	–
	Range	4–20								
1	Median (IQR)	3 (3–7)	-2.701	**0.007**	1.5 (1–4) 0–6	– 2.965	**0.003**	3 (3–7) 2–20	– 3.427 ≠	**0.001**
	Range	2–20								
2	Median (IQR)	2 (1–6)	-2.684	**0.007**	0 (0–1) 0–6	-2.949	**0.003**	2 (1–6) 0–20	– 3.424 ≠	**0.001**
	Range	0–20								

^*^Wilcoxon test

There was no statistical significant difference between both groups A and B regarding median (IQR) nail matrix NA*P*SI score at 0 (*P* = 0.254), 1 (*P* = 0.432) and 2 months (*P* = 0.448). There was no statistical significant difference between both groups A and B regarding median (IQR) nail bed NAPSI score at 0 (*P* = 0.604), 1 (*P* = 0.442) and 2 months (*P* = 0.891). There was no statistical significant difference regarding total NAPSI score between both group A and B at 0 (*P* = 0.271), 1(*P* = 0.513) and 2 months (*P* = 0.647) (Table 5).

**Table 5:** Comparison between group A and B regarding median (IQR) nail matrix, nail bed and total NAPSI score at 0, 1, and 2 months

Month/s		Total Nail matrix score		Test value	*P*-value	Total Nail bed score		Test value	*P*-value	Total NAPSI score		Test value	*P*-value
		Group A	Group B			Group A	Group B			Group A	Group B		
0	Median (IQR)	9 (6–13)	5 (4–9)	–1.140 ≠	0.254	4 (2–8) 2–15	3.5 (2–6) 2–9	–0.519 ≠	0.604	8 (3.5–13) 2–16	6.5 (3.5–9) 2–20	–1.101 ≠	0.271
	Range	3–16	4–20										
1	Median (IQR)	4.5 (3–10)	3 (3–7)	–0.786 ≠	0.432	2 (1–5) 0–7	1.5 (1–4) 0–6	–0.769 ≠	0.442	5 (2.5–7) 1–16	4 (2–6) 0–20	–0.654 ≠	0.513
	Range	2–16	2–20										
2	Median (IQR)	1 (1–9)	2 (1–6)	–0.758 ≠	0.448	0 (0–2) 0–6	0 (0–1) 0–6	–0.137 ≠	0.891	1 (0–4) 0–16	2 (0.5–3.5) 0–20	–0.457 ≠	0.647
	Range	0–16	0–20										

^*^ Mann–Whitney test

There was a high statistical significant decrease in median (IQR) NAPSI score of nail pitting (*P* = 0.001), onycholysis (*P* = 0.000) and oil drops (*P* = 0.008) nail symptoms at 1 and 2 months in group A. However, there was no significant statistical decrease in median (IQR) NAPSI score of nail ridging (*P* = 1.000) and subungual hyperkeratosis (*P* = 0.156) nail symptoms at 1 and 2 months in the same group (Table 6).

**Table 6:** Comparison between median (IQR) NAPSI scores for each of nail matrix and nail bed symptoms in group A at 0, 1, and 2 months

Nail matrix		Group A			Test value ≠	*P*-value	P1 (0 Vs 1)	P2 (0 Vs 2)	P3 (1 Vs 2)
		0	1	2					
Nail pitting	Median (IQR)	7 (4–10)	4 (3–5)	1 (0–1)	14.000	**0.001 (HS)**	0.016	0.018	0.018
	Range	4–11	1–5	0–2					
Nail ridging	Median (IQR)	14 (9–16)	14 (9–16)	14 (9–16)	0.000	1.000 **(NS)**	1.000	1.000	1.000
	Range	9–16	9–16	9–16					
**Nail bed**									
Onycholysis	Median (IQR)	4.5 (3–7)	2 (1–3.5)	0 (0–0.5)	15.548	**0.000 (HS)**	0.011	0.012	0.017
	Range	2–13	0–5	0–2					
Subungual									
hyperkeratosis	Median (IQR)	7 (2–8)	7 (2–7)	6 (2–6)	3.714	0.156 **(NS)**	0.317	0.180	0.157
	Range	2–8	2–7	2–6					
Oil drops	Median (IQR)	2 (2–6)	1 (1–2)	0 (0–0)	9.579	**0.008 (HS)**	0.041	0.039	0.059
	Range	2–7	0–3	0–1					

^*^Friedman test, adjusted Wilcoxon rank test

In group B, there was a high statistical decrease in median IQR of nail pitting (*P* = 0.000), onycholysis (*P* = 0.000) and significant statistical decrease in median IQR of oil drops (*P* = 0.050) nail symptoms at 1 and 2 months. However, there was no significant statistical decrease in median IQR of nail ridging (*P* = 1.000), subungual hyperkeratosis (*P* = 1.000) at 1 and 2 months in the same group (Table 7).

**Table 7:** Comparison between Median (IQR) NAPSI score for each of nail matrix and bed symptoms in group B at 0, 1, and 2 months

Nail matrix		Group B			Test value	*P*-value	P1 (0 Vs 1)	P2 (0 Vs 2)	P3 (1 Vs 2)
		0	1	2					
Nail pitting	Median (IQR)	4 (4–7)	3 (2–3.5)	1.5 (0.5–2)	15.548	**0.000** (**HS**)	0.011	0.011	0.016
	Range	3–12	1–7	0–2					
Nail ridging	Median (IQR)	6 (6–20)	6 (6–20)	6 (6–20)	0.000	1.000 (**NS**)	1.000	1.000	1.000
	Range	6–20	6–20	6–20					
**Nail bed**									
Onycholysis	Median (IQR)	3 (2–5)	1 (1–3)	0 (0–1)	17.176	**0.000** (**HS**)	0.007	0.007	0.016
	Range	2–9	0–6	0–1					
Subungual hyperkeratosis	Median (IQR)	4 (2–6)	4 (2–6)	4 (2–6)	0.000	1.000 (**NS**)	1.000	1.000	1.000
	Range	2–6	2–6	2–6					
Oil drops	Median (IQR)	3 (3–4)	1 (1–1)	0 (0–0)	6.000	**0.050 (S)**	0.102	0.102	0.083
	Range	3–4	1–1	0–0					

^*^Friedman test, adjusted Wilcoxon rank test

There was no statistical difference in median (IQR) NAPSI scores of different nail bed and matrix symptoms between group A and B at 0, 1, and 2 months (Table 8).

**Table 8:** Comparison between group A and B regarding the median (IQR) NAPSI scores of different nail symptoms at 0, 1, and 2 months

		Absolute values		Test value	*P*-value	Sig
		Group A	Group B			
NAIL MATRIX SYMPTOMS						
Nail pitting						
0	Median (IQR)	7 (4–10)	4 (4–7)	–1.195 ≠	0.232	NS
	Range	4–11	3–12			
1	Median (IQR)	4 (3–5)	3 (2–3.5)	–1.008 ≠	0.313	NS
	Range	1–5	1–7			
2	Median (IQR)	1 (0–1)	1.5 (0.5–2)	–1.225 ≠	0.221	NS
	Range	0–2	0–2			
Nail ridging						
0	Median (IQR)	14 (9–16)	6 (6–20)	–0.664 ≠	0.507	NS
	Range	9–16	6–20			
1	Median (IQR)	14 (9–16)	6 (6–20)	–0.664 ≠	0.507	NS
	Range	9–16	6–20			
2	Median (IQR)	14 (9–16)	6 (6–20)	–0.664 ≠	0.507	NS
	Range	9–16	6–20			
NAIL BED SYMPTOMS						
Onycholysis						
0	Median (IQR)	4.5 (3–7)	3 (2–5)	–0.782 ≠	0.434	NS
	Range	2–13	2–9			
1	Median (IQR)	2 (1–3.5)	1 (1–3)	–0.345 ≠	0.730	NS
	Range	0–5	0–6			
2	Median (IQR)	0 (0–0.5)	0 (0–1)	–0.181 ≠	0.857	NS
	Range	0–2	0–1			
Subungual						
hyperkeratosis						
0	Median (IQR)	7 (2–8)	4 (2–6)	–0.886 ≠	0.376	NS
	Range	2–8	2–6			
1	Median (IQR)	7 (2–7)	4 (2–6)	–0.899 ≠	0.369	NS
	Range	2–7	2–6			
2	Median (IQR)	6 (2–6)	4 (2–6)	–0.471 ≠	0.637	NS
	Range	2–6	2–6			
Oil drops						
0	Median (IQR)	2 (2–6)	3 (3–4)	–0.461 ≠	0.645	NS
	Range	2–7	3–4			
1	Median (IQR)	1 (1–2)	1 (1–1)	–0.512 ≠	0.608	NS
	Range	0–3	1–1			
2	Median (IQR)	0 (0–0)	0 (0–0)	–0.775 ≠	0.439	NS
	Range	0–1	0–0			

^*^Mann–Whitney test

There was no statistical significant difference between both groups regarding pain (*P* = 1.000) and bleeding (*P* = 1.000) side effects during sessions. However, there was a high statistical significant difference between both groups regarding nail yellow discoloration (*P* = 0.000), as all group A showed nail yellowish discoloration which lasted for 2 days after each session, in contrast, group B didn’t show any nail yellowish discoloration at all.

There was no statistical significant difference regarding patient satisfaction (*P* = 0.980) between group A and B (Table 9).

**Table 9:** Comparison between group A and B regarding level of patients’ satisfaction

Patients satisfaction	Group A	Group B	Test value	*P*-value
	No. (%)	No. (%)		
Not at all	5 (25.0%)	6 (30.0%)	0.182*	0.980
Partially satisfied	1 (5.0%)	1 (5.0%)		
Satisfied	6 (30.0%)	5 (25.0%)		
Completely	8 (40.0%)	8 (40.0%)		

^*^Chi-square test

## Discussion

The prevalence of nail involvement in psoriasis patients varies between 15 and 79% [12]. The most common sign in nail psoriasis is pitting, occurring in almost 70% of patients, followed by onycholysis [13].

Treating nail psoriasis is often a time-consuming challenge with an unsecure outcome. Response to the treatment is variable and relapses are common. Therapeutic options include topical treatments, intra-lesional treatments, and systemic treatments [14].

Systemic treatments have multiple systemic side effects that affect other body organs, so it is used only in cases of nail psoriasis with severe skin psoriasis or psoriatic arthritis. Lack of the patient compliance makes intra-lesional therapies not the best choice for the treatment of nail psoriasis because of its side effects as short-term paraesthesia and focal pain that may last for several months [15].

Penetration of a topical medication into the site of psoriatic nail inflammation, the nail bed or the nail matrix is essential to achieve therapeutic concentrations. Given the anatomical structure and physical characteristics of the nail, it is difficult, or impossible, for anti-psoriatic agents to penetrate through the nail plate to the site of psoriatic inflammation [16].

Laser therapy has proved to be effective and safe therapy for nail psoriasis, either alone or in combination with other modalities, being beneficial especially with topical treatments. Laser can help to improve this resistant form of psoriasis with high patient’s satisfaction. Vascular lasers are supposed to exert its effect on angiogenesis and vascularity within the psoriatic nail. Several case reports and clinical studies have been reported; however, the results are rather contradictory. While some authors claim effects mainly on nail bed psoriasis, others report more positive results on nail matrix psoriasis, or even negative effects on nail bed psoriasis [17].

In the current study, we aimed to evaluate and compare the efficacy, safety and side effects of MTX (group A) and calcipotriol plus betamethasone two-compound formula (group B) when applied topically to psoriatic nails after their exposure to fractional CO_2_ laser which creates pores to facilitate the penetration and delivery of both medications through the nail plate to reach the site of psoriatic inflammation. To our knowledge, this study is the first to evaluate these treatment modalities within each of the included patients. This allows comprehensive and solid assessment of these modalities after excluding the effect of patient's age, sex, and other individual variations that may influence the response to treatment when the study design compares different treatment modalities in different patients.

At the end of the treatment sessions, both studied groups showed a marked clinical and statistical improvement in the nail matrix, nail bed and total NAPSI score. However, there was no statistical significant difference in the median (IQR) NAPSI score between both nail matrix and nail bed in both groups.

These results of group A confirm those of Nguyen and Banga (2018) and Alakad et al. (2022) who concluded that fractional ablative laser was found to improve the transungual delivery of MTX [8, 9].

The results of group B agree with Rigopoulos et al. (2009) who studied the effect of topical calcipotriol plus betamethasone two-compound ointment alone on psoriatic nails without any fractional CO_2_ laser sessions before the drug application. Their study included 22 patients with nail psoriasis who were instructed to apply the medication once daily at bedtime onto the nail plate folds and hyponychium of the affected nails for 12 weeks. The results showed reduction of the mean total NAPSI score at the end of the 12 weeks by 72% of its value at the baseline. While, our study results showed improvement in the median (IQR) total NA*P*SI score by 81% at the end of our treatment protocol within a period of 8 weeks only (*P* = 0.001), and this signifies the importance of fractional CO_2_ laser in improving drug delivery, drug efficacy and shortening of the treatment duration [10].

In comparison between both groups (A) and (B) at the end of the treatment sessions, there was no statistical significant difference between both groups regarding median (IQR) NAPSI score of nail matrix, nail bed, or total NAPSI score. These findings suggest that both topical preparations are nearly equally effective.

On the analysis of the different nail signs at the end of the treatment sessions, we noticed that there were some nail signs that showed marked improvement in both groups as onycholysis, nail pitting and oil drops, while other symptoms as nail ridging and subungual hyperkeratosis, did not show any improvement at all.

These results partially agree with Rigopoulos et al., (2009), whose study results showed significant decrease in the mean NAPSI score of onycholysis, subungual hyperkeratosis, moderate decrease in oil drop nail symptom and the least reduction in the mean NAPSI score was with nail pitting [10].

This difference between our study and Rigopoulos et al., (2009) regarding the improvement in subungual hyperkeratosis can be referred to the thickening of the nail bed which needed more time for the fractional CO_2_ laser to penetrate and facilitate drug delivery, so application of topical medications once after each of the four fractional CO_2_ laser sessions wasn’t effective. While, Rigopoulos et al., (2009) applied the medication once daily for 12 weeks which might reduce the nail bed thickness by time and maximize the drug delivery through the nail plate by the end of the treatment course [10].

In nail pitting, our study showed more improvement than Rigopoulos et al., (2009) as the mission of fractional CO_2_ laser here was easier to penetrate the nail plate and facilitate the drug delivery into the site of psoriatic inflammation in the nail matrix in a faster and more effective way [10].

In comparison between both groups (A) and (B) regarding the improvement in nail signs at the end of the treatment sessions, there was no statistical significant difference in median (IQR) NAPSI scores of different nail matrix and bed signs.

As regards patient’s satisfaction, more than 50% of patients in both groups were satisfied with the treatment, and this adds to the significant improvement observed on comparing the NAPSI scores before and after treatment in both groups.

On evaluation of our treatment protocol’s side effects, we noticed that during the fractional CO_2_ laser sessions, there were 50% of the patients in each group experienced pain which lasted for one day only and 25% of the patients in each group showed nail bleeding that stopped immediately after the sessions. Both pain and bleeding side effects can be referred to the high energy applied on the nails by fractional CO_2_ laser to facilitate the penetration of nail plates. However, in group A only, we noticed yellowish discoloration of the nails treated with topical MTX which caused a temporal nail staining by its yellow color for 2 days only. These side effects didn’t show any impact on patients’ satisfaction as it was self-limited and lasted for a very short period.

Thus, we can say from our observations in the present work that combined fractional CO_2_ laser with either topical MTX or topical betamethasone plus calcipotriol two-compound formula is effective treatment for nail psoriasis.

## Author's contribution statement

El-bana MG was responsible for the practical part. Afify AA and Shaheen MA advised him through the sessions. Afify AA was the one who prepared the manuscript. All the authors provided final approval of the version to be published.

## Data Availability

Data is available upon special request.
